# Artificial Intelligence Combined With Big Data to Predict Lymph Node Involvement in Prostate Cancer: A Population-Based Study

**DOI:** 10.3389/fonc.2021.763381

**Published:** 2021-10-14

**Authors:** Liwei Wei, Yongdi Huang, Zheng Chen, Hongyu Lei, Xiaoping Qin, Lihong Cui, Yumin Zhuo

**Affiliations:** ^1^ Department of Urology, the First Affiliated Hospital of Jinan University, Guangzhou, China; ^2^ College of Mathematics and Physics, Beijing University of Chemical Technology, Beijing, China

**Keywords:** prostate cancer, machine learning, lymph node involvement, predictive model, SEER database

## Abstract

**Background:**

A more accurate preoperative prediction of lymph node involvement (LNI) in prostate cancer (PCa) would improve clinical treatment and follow-up strategies of this disease. We developed a predictive model based on machine learning (ML) combined with big data to achieve this.

**Methods:**

Clinicopathological characteristics of 2,884 PCa patients who underwent extended pelvic lymph node dissection (ePLND) were collected from the U.S. National Cancer Institute’s Surveillance, Epidemiology, and End Results (SEER) database from 2010 to 2015. Eight variables were included to establish an ML model. Model performance was evaluated by the receiver operating characteristic (ROC) curves and calibration plots for predictive accuracy. Decision curve analysis (DCA) and cutoff values were obtained to estimate its clinical utility.

**Results:**

Three hundred and forty-four (11.9%) patients were identified with LNI. The five most important factors were the Gleason score, T stage of disease, percentage of positive cores, tumor size, and prostate-specific antigen levels with 158, 137, 128, 113, and 88 points, respectively. The XGBoost (XGB) model showed the best predictive performance and had the highest net benefit when compared with the other algorithms, achieving an area under the curve of 0.883. With a 5%~20% cutoff value, the XGB model performed best in reducing omissions and avoiding overtreatment of patients when dealing with LNI. This model also had a lower false-negative rate and a higher percentage of ePLND was avoided. In addition, DCA showed it has the highest net benefit across the whole range of threshold probabilities.

**Conclusions:**

We established an ML model based on big data for predicting LNI in PCa, and it could lead to a reduction of approximately 50% of ePLND cases. In addition, only ≤3% of patients were misdiagnosed with a cutoff value ranging from 5% to 20%. This promising study warrants further validation by using a larger prospective dataset.

## Introduction

Prostate cancer (PCa) is the most common type of malignant tumor in American men and accounts for nearly 15% of all cancer cases. Recurrence and metastasis are the most common causes of death in PCa patients (1). Radical prostatectomy (RP) is the gold-standard treatment for patients with PCa and those with either organ-confined or locally advanced PCa can benefit from it (2–4). Because of the biological characteristics of this disease and its response to effective treatment, patients with PCa generally have an excellent long-term prognosis with the vast majority of patients surviving over 5 years (5). However, if a PCa patient is diagnosed with lymph node involvement (LNI), the probability of tumor recurrence will increase, and the prognosis will deteriorate significantly (6, 7).

For a lymph node (LN)-positive PCa patient, extended pelvic lymph node dissection (ePLND) can often offer a better cancer-specific outcome either with or without adjuvant androgen deprivation therapy (ADT) (8, 9). As LNI is a significant component in the prognosis of a patient, it can influence any clinical decisions made by the surgeon. Before surgery, it is essential to know the precise clinical status of LNI in patients with PCa. To this end, researchers have reported several approaches and tools that can help the clinician to estimate the occurrence of LNI, including prostate-specific membrane antigen-positron emission tomography (PSMA-PET) scans, multiparametric magnetic resonance imaging (mpMRI), and the use of some emerging molecular biomarkers (10–13). However, these imaging techniques are not accurate and most of the molecular biomarkers are unproven. Therefore, the most commonly used tools available are Briganti, Partin, and Memorial Sloan Kettering Cancer Center (MSKCC) nomograms which have an accuracy of less than 80% (14–17). Machine learning (ML) is an emerging intersection approach involving many fields that allows for accurate prediction of outcomes from multiple unrelated datasets, which would otherwise be discrete and difficult to associate (18).

With the rapid development of evidence-based medicine, vast and complex medical datasets need more advanced techniques for their interpretation, and ML is becoming a promising option for the diagnosis and prognosis/prediction of many diseases (19, 20). In addition, ML has demonstrated excellent performance of predictive abilities and a good potential application in several areas of medicine (21). Our goal was to develop a new decision-support ML model based on big data for predicting the risk of LNI in PCa patients. We used area under the curves (AUCs), calibration plots, and decision curve analysis (DCA) to evaluate the performance of the model. We further validated the accuracy of our ML model by using a validation set.

## Material and Methods

### Data Source and Study Population

In this study, we used the Surveillance, Epidemiology, and End Results (SEER) database (https://seer.cancer.gov/) from the National Cancer Institute, a freely available cancer registry in the United States. We obtained permission to access the files of the SEER database and all authors followed the SEER database regulations throughout the study. No personally identifiable information was used in this study and informed consent was not necessary from individual participants. The Medical Ethics Committee at Jinan University’s First Affiliated Hospital examined and approved this work.

**Figure 1:**
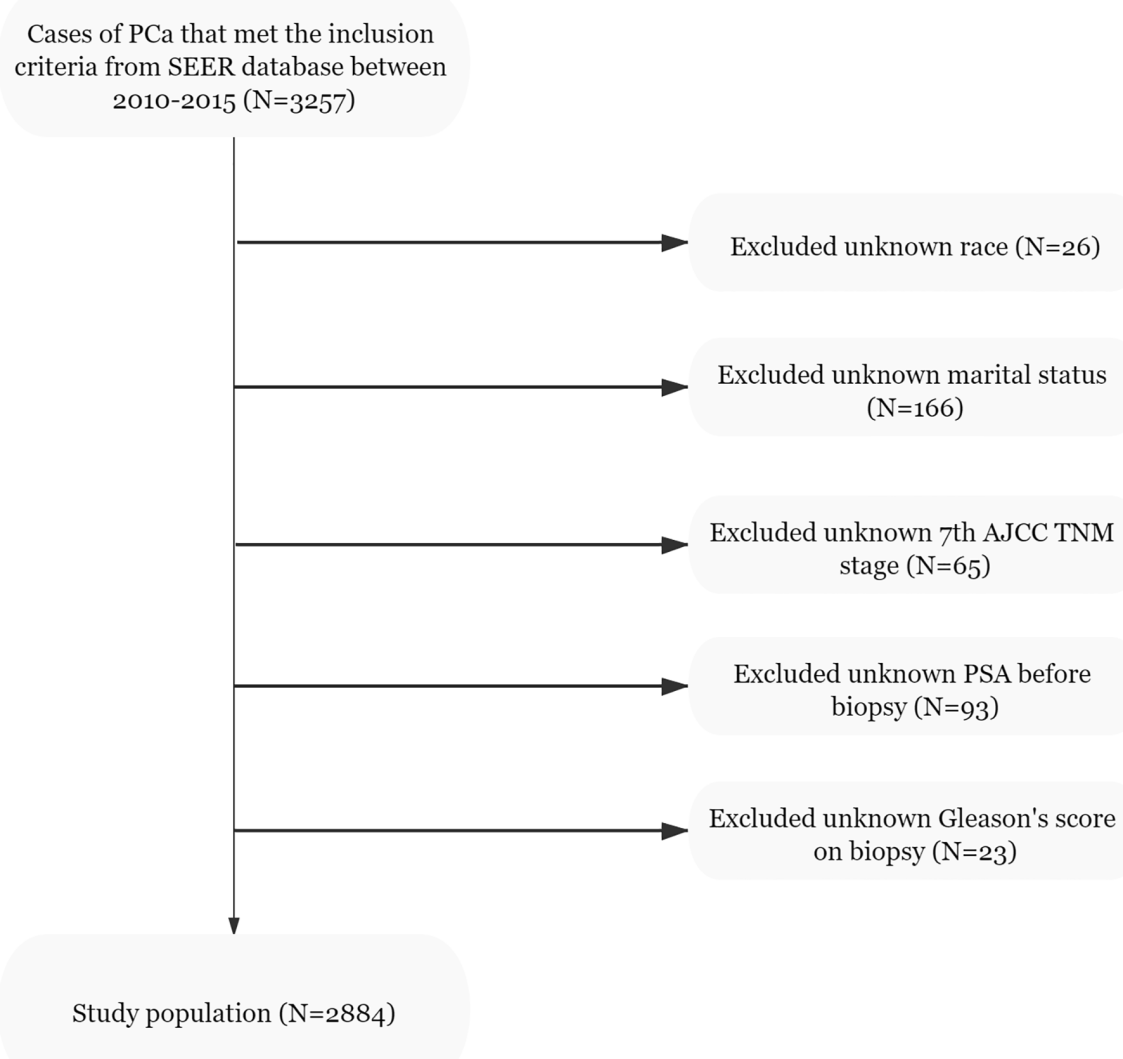
Flowchart of the study population selected from the SEER database, based on the inclusion and exclusion criteria outlined above; 2,884 patients were included in this study.

Data of the patients were downloaded from the SEER 18 Regs Research Data Nov 2018 Sub (1975–2016) by using the SEER*Stat 8.3.9.1 software. The selection criteria included men aged 35–90 years who were diagnosed with histologically confirmed prostatic adenocarcinoma (site code C61.9, morphology code 8140/3). The patients were diagnosed between 2010 and 2015 and PCa was the first malignant tumor found. All cases were treated with radical prostatectomy (RP) and ePLND without neoadjuvant systematic therapy. However, as the data obtained did not include ePLND as defined by anatomical location, we referred to the literature and defined this as more than or equal to 10 LNs being removed from the patients (22, 23). The T stage of the tumors was derived from preoperative examination and postoperative specimens. The number of needle cores examined was between 4 and 24, and the autopsy cases were excluded. The downloaded data of the patients were obtained from 3,257 cases. The exclusion criteria were as follows: unknown information from the American Joint Committee on Cancer seventh TNM stage, race, marital status, prostate-specific antigen (PSA) levels before biopsy, and the absence of Gleason scores (GS). From the 3,257 initial cases considered, 2,884 patients remained in the final cohort for use in model development. The population data selection procedure used is shown in **Figure 1**. The validation set was derived from the same SEER database and cases were diagnosed in 2016. Besides the above criteria, an extra one from the Briganti nomogram was chosen: patients with PSA >50 ng/ml were excluded from the validation set. A total of 535 patients were included in the analysis as the validation set.

### Variable Selection

Several readily available clinical and demographic characteristics were chosen as independent variables for analysis. Since the SEER database does not include tumor size based on preoperative imaging, this parameter is prone to some errors. However, the tumor size of postoperative specimens in most patients did not differ significantly from the tumor size evaluated by imaging, and therefore, we considered this error to be within an acceptable range (24–27). At last, eight demographic and clinicopathological variables, namely, age at diagnosis, race, marital status, T stage, tumor size, PSA levels before biopsy, GS on biopsy, and percentage of positive cores (PPC), were selected as independent variables for analysis.

### Model Establishment and Development

**Figure 2:**
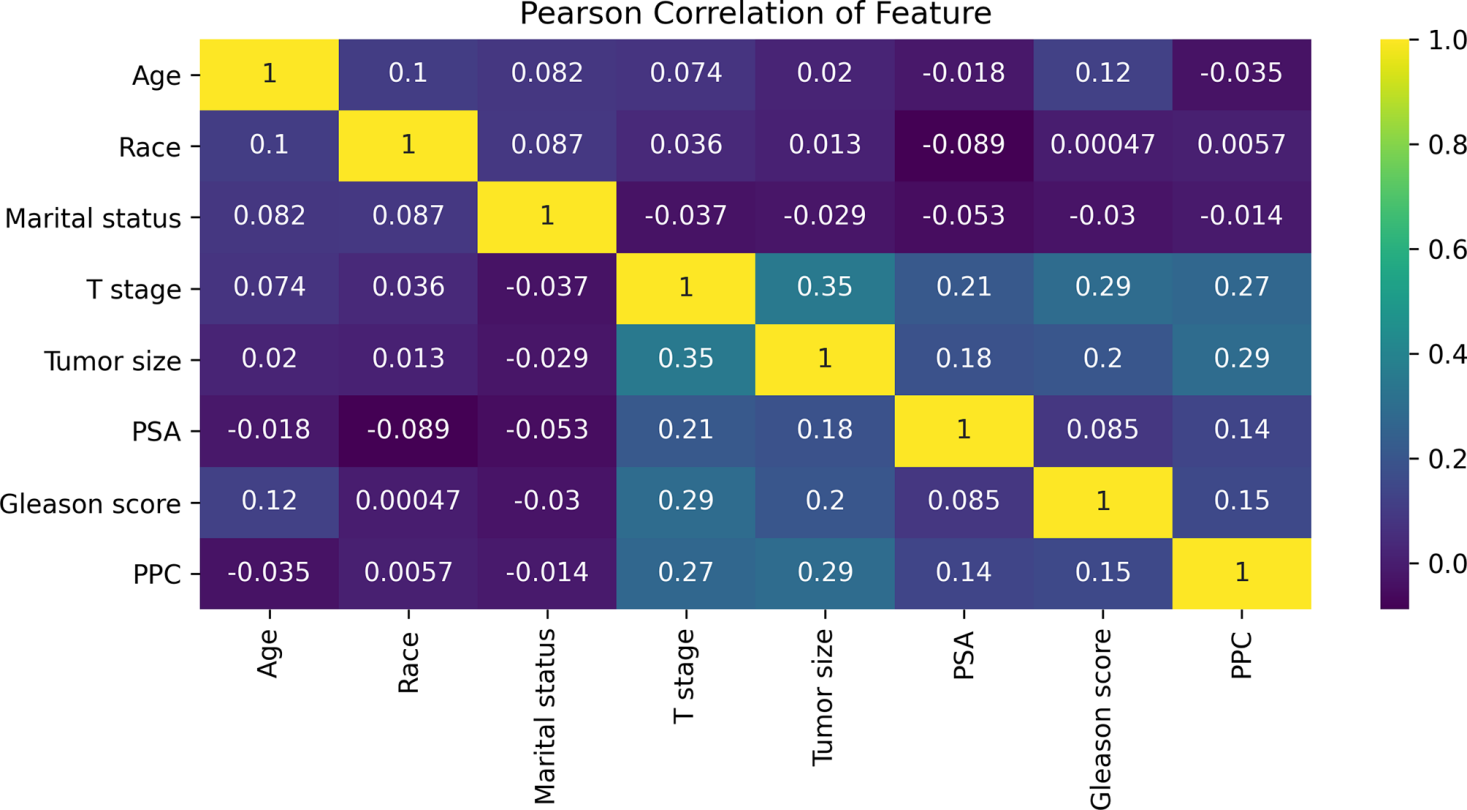
The results of Pearson correlation analysis between all the variables. The heat map shows the correlation between the variables. Abbreviations: PSA, prostate-specific antigen; PPC, percentage of positive cores.

All statistical analyses in the study were performed using SPSS (version 22, IBM SPSS Software Foundation) and Python (version 3.8.1, Python Software Foundation). All variables were tested for Pearson correlations and the results are presented as a heat map (**Figure 2**). All patients studied were randomly divided into a training set and a test set at a ratio of 7:3 (**Table 1**). The chi-square test was used to analyze the differences between the training and test sets. The training set was used to establish the extreme gradient boosting (XGB) and multivariate logistic regression (MLR) models, and the test set was then applied to evaluate both of them. We used 600 trees in XGB to build the ML model. For MLR, we used an entry variable selection method to establish the model. Then, the 5%, 10%, 15%, and 20% cutoff values of the XGB and MLR models were calculated, and these were used respectively to test their clinical value for directing the possible treatment options.

**Table 1:** Clinical and pathological characteristics of the training and test sets.

Variables	Training set		Test set		*p*-value
	NLNI (n = 1,787) (%)	LNI (n = 231) (%)	NLNI (n = 753) (%)	LNI (n = 113) (%)	
Age at diagnosis					0.072
<62	801 (44.8)	111 (48.1)	362 (48.1)	61 (46.0)	
≥62	986 (55.2)	120 (51.9)	391 (51.9)	52 (54.0)	
Race					0.578
Black	203 (11.3)	24 (10.4)	99 (13.2)	15 (13.4)	
Other	138 (7.7)	13 (5.6)	37 (4.9)	8 (7.0)	
White	1,446 (81.0)	194 (84.0)	617 (81.9)	90 (79.6)	
Marital status					0.683
Unmarried	178 (9.9)	33 (14.3)	79 (10.5)	16 (14.2)	
Married	1,609 (90.1)	198 (85.7)	674 (89.5)	97 (85.8)	
T stage					0.371
T1	1 (0.0)	0 (0.0)	0 (0.0)	0 (0.0)	
T2	1,092 (61.2)	26 (11.2)	458 (60.8)	12 (10.6)	
T3	693 (38.8)	199 (86.1)	291 (38.7)	96 (84.9)	
T4	1 (0.0)	6 (2.7)	4 (0.5)	5 (4.5)	
Tumor size					0.415
0~10 mm	260 (14.5)	8 (3.5)	104 (13.8)	3 (2.7)	
10~20 mm	812 (45.4)	51 (22.1)	344 (45.7)	22 (19.5)	
20~30 mm	442 (24.7)	69 (29.9)	186 (24.7)	39 (34.5)	
>30 mm	273 (15.4)	103 (44.5)	119 (15.8)	49 (43.3)	
PSA before biopsy					0.859
0~4 ng/ml	205 (11.5)	15 (6.5)	82 (10.9)	6 (5.3)	
4~10 ng/ml	1,144 (64.0)	102 (44.1)	495 (65.7)	43 (38.1)	
10~20 ng/ml	316 (17.7)	57 (24.7)	139 (18.5)	29 (25.7)	
>20 ng/ml	122 (6.8)	57 (24.7)	37 (4.9)	35 (30.9)	
Primary biopsy Gleason score					0.223
≤6	323 (18.1)	8 (3.5)	112 (14.9)	5 (4.4)	
7	1073 (60.0)	102 (44.2)	446 (59.2)	50 (44.2)	
8	249 (13.9)	57 (24.7)	127 (16.9)	29 (25.7)	
≥9	142 (8.0)	64 (27.6)	68 (9.0)	29 (25.7)	
Positive biopsy percentage					0.650
0~25%	544 (30.4)	27 (11.7)	233 (30.1)	9 (8.0)	
25~50%	668 (37.4)	69 (29.9)	279 (37.1)	37 (32.7)	
50~75%	359 (20.1)	55 (23.8)	145 (19.3)	26 (23.0)	
75~100%	216 (12.1)	80 (34.6)	96 (13.5)	41 (36.3)	

### Model Improvement

**Figure 3:**
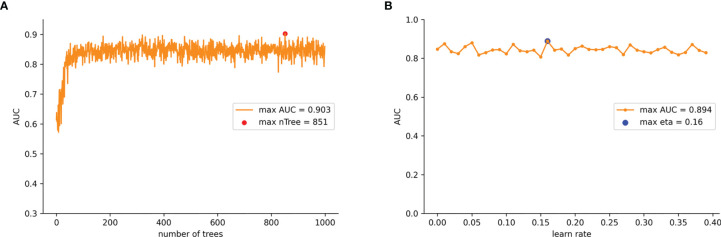
**(A)** AUC values for nTree values from 1 iterates to 1,000 in the improved XGB model. **(B)** AUC values for learn rate from 0.01 iterates to 0.4 in the improved XGB model.

To ensure that the model was stable, a 10-fold cross-validation was adopted to evaluate the predictive capability of the model. The training set was randomly divided into 10 groups. In each iteration of 10-fold cross-validation, nine groups were randomly selected for training, and the remaining group was used as the test set. This means that each group was chosen as the test set in turn, which ensured that the evaluation results were not accidental. Then we averaged the results of the 10 evaluations in order to reduce the errors caused by any unreasonable selections made in the test set. To achieve the overall optimum value in the XGB model, we used the learning curve method to find the optimal parameters. The learning curve is shown in **Figure 3** where the abscissa axis represents the number of trees and different learning rates, and the ordinate axis represents the average AUC of the 10-fold cross-validation. The final optimal parameter combination was as follows: the number of trees (“*n* tree”) = 851 (**Figure 3A**), the learning rates (“eta”) = 0.16 (**Figure 3B**), the maximum length from the root node to leaf node (“max depth”) = 6, the sum of weights of the minimum leaf node samples (“min child weight”) = 1, and the L2 regularization parameters (“reg lambda”) = 120. All other parameters were selected as default values for the calculations.

### Evaluation of Model Performance

The performance analysis used comprised of three components. Firstly, model discrimination was quantified with receiver operating characteristic (ROC) curve analysis, and its predictive accuracy was assessed with the AUCs obtained. Secondly, we used calibration plots to evaluate the performance of the model, which indicated the calibration and how far the predictions of the model deviated from the actual event. Thirdly, clinical usefulness and net benefits were assessed with DCA which could estimate the net benefit by calculating the difference between the true- and false-positive rates and weighted these by the odds of the selected threshold probability of risks involved. Also, additional ML algorithms such as decision tree (DT) and support vector machine (SVM) were introduced for comparison. ROC curves and calibration plots were used to further evaluate the appropriateness and generalizability of our model.

## Results

### Demographic and Clinicopathological Characteristics

A total of 2,884 PCa patients were analyzed in this study. Three hundred forty-four patients had LNI (11.9%) and 2,540 (88.1%) did not have and these were classified as none LNI (nLNI). All patients were completely randomized with a ratio of 7:3 into a training set (*n* = 2,018) and a test set (*n* = 866). The demographic and clinicopathological variables of the patients are detailed in **Table 1**.

### Model Analysis and Variable Feature Importance of the Prediction

**Figure 4:**
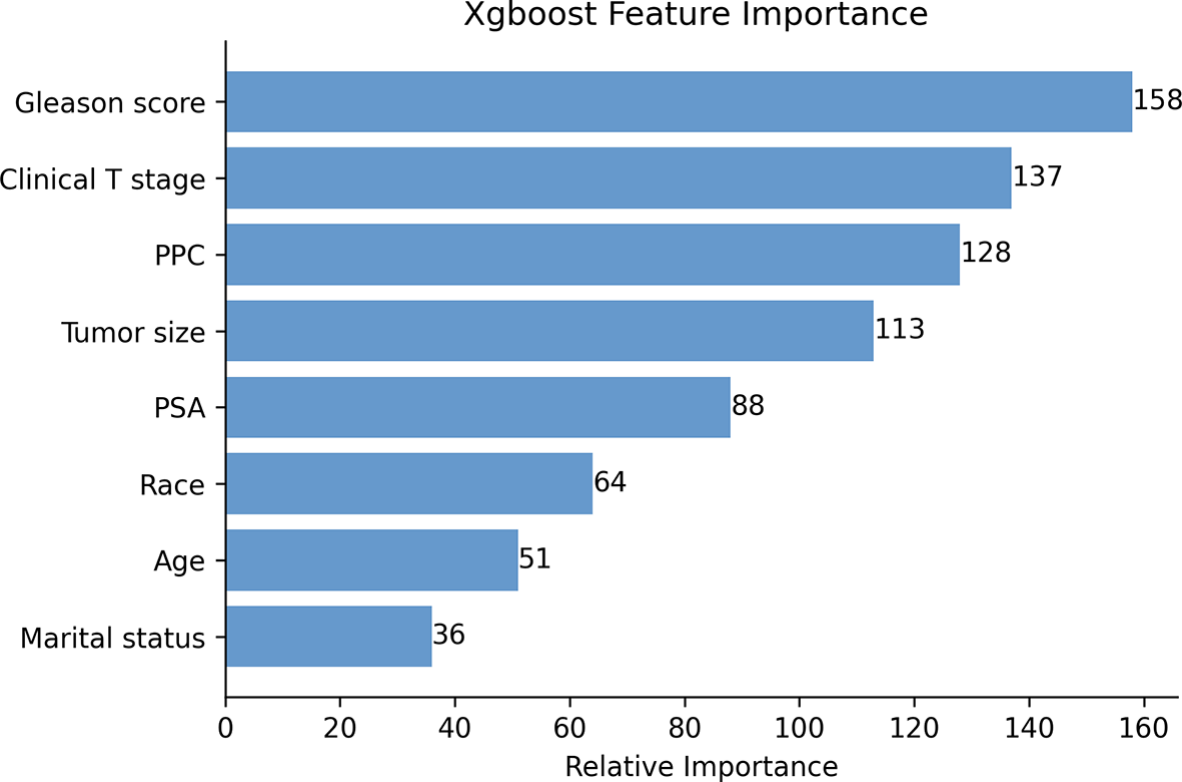
The XGB model was used to calculate the importance of each feature. The bar chart depicts the relative significance of the variables.

The Pearson correlation analysis was performed for all the factors. A correlation heat map showed weak correlations between several clinicopathological variables (T stage, tumor size, PSA, GS, and PPC), and there was a moderate correlation between T stage and tumor size (**Figure 2**). For the MLR model, five of the eight variables were identified as independent risk factors, namely, T stage (*p* < 0.001), tumor size (*p* < 0.001), PSA before biopsy (*p* < 0.001), GS (*p* < 0.001), and PPC (*p* = 0.006) (**Table 2**). For the XGB model, we identified the feature of importance by the size of the gain value for each variable, with the higher values indicating more importance for the prediction target: GS (158 points), T stage (137 points), PPC (128 points), tumor size (113 points), PSA (88 points), race (64 points), age at diagnosis (51 points), and marital status (36 points) (**Figure 4**).

**Table 2:** Multivariable logistic regression model with the entered variable selection.

Variables	OR (95% CI)	*p*-value
Age at diagnosis		
<62	Reference	
≥62	0.771 (0.592–1.003)	0.053
Race		
Black	Reference	
Other	1.047 (0.551–1.990)	0.889
White	1.251 (0.826–1.893)	0.290
Marital status		
Unmarried	Reference	
Married	0.883 (0.598–1.305)	0.533
T stage		
T1	–	
T2	Reference	
T3	6.363 (4.409–9.181)	<0.001
T4	32.343 (9.977–104.839)	<0.001
Tumor size		
0~10 mm	Reference	
10~20 mm	1.376 (0.691–2.737)	0.363
20~30 mm	2.436 (1.233–4.816)	0.010
>30 mm	4.033 (2.044–7.957)	<0.001
PSA before biopsy		
0~4 ng/ml	Reference	
4~10 ng/ml	1.123 (0.666–1.895)	0.663
10~20 ng/ml	1.785 (1.026–3.105)	0.040
>20 ng/ml	4.118 (2.310–7.338)	<0.001
Gleason’s score		
≤6	Reference	
7	2.414 (1.315–4.431)	0.004
8	4.393 (2.321–8.315)	<0.001
≥9	5.696 (2.986–10.867)	<0.001
Positive biopsy percentage		
0~25%	Reference	
25~50%	1.679 (0.929–2.294)	0.662
50~75%	1.961 (0.961–2.379)	0.083
75~100%	4.368 (2.413–7.533)	0.006

### Model Performance

**Figure 5:**
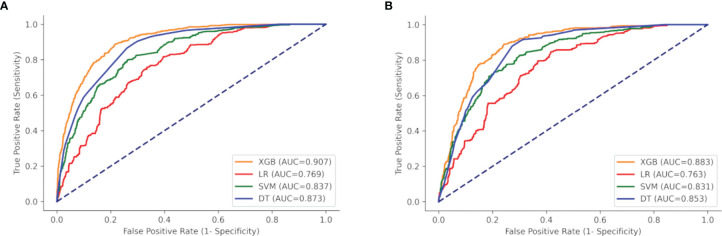
ROC curves of the four models: XGB, SVM, DT, and LR. **(A)** Training set, and **(B)** test set. XGBoost, extreme gradient boosting; SVM, support vector machine; DT, decision tree; LR, multivariate logistic regression.

ROC curves, calibration plots, and DCA for the training set (*n* = 2,018) and the test set (*n* = 866) were constructed in order to determine the accuracy of our models. The XGB model had the best performance in both training and test sets (AUC = 0.907 and 0.883, respectively), compared with SVM (AUC = 0.837 and 0.831, respectively), DT (AUC = 0.873 and 0.853, respectively), and MLR (AUC = 0.769 and 0.763, respectively) (**Figures 5A, B**). The sensitivity, specificity, and cutoff values of the predictions of the two models were also calculated for the patients having LNI or not. In addition, the predictive accuracy and error when the patients were predicted to be at risk of LNI are given in **Table 3**. The results showed that whether 5%, 10%, 15%, or 20% was chosen as the cutoff value, the XGB model was better than the MLR model in reducing omissions and avoiding overtreatment of patients, with a lower false-negative rate and a higher percentage of ePLND avoided. With a 5%–20% cutoff value, the XGB model could keep the risk of missing patients below 3% (1.2%–2.9%).

**Table 3:** Analyses of the cutoff values of the XGB model based on the test set.

Treatment option	False positive, *n*/*N* (%)	False negative, *n*/*N* (%)	True positive, *n*/*N* (%)	True negative, *n*/*N* (%)
5% cutoff				
XGB	287/438 (65.5)	5/428 (1.2)	151/438 (34.5)	423/428 (98.9)
LR	459/630 (72.9)	6/236 (2.5)	171/630 (27.1)	230/236 (97.5)
10% cutoff				
XGB	186/322 (57.8)	9/544 (1.7)	136/322 (42.2)	535/544 (98.3)
LR	244/361 (67.6)	20/505 (4.0)	117/361 (32.4)	485/505 (96.0)
15% cutoff				
XGB	133/262 (50.8)	15/604 (2.5)	129/262 (49.2)	589/604 (97.5)
LR	139/222 (62.6)	34/644 (5.3)	83/222 (37.4)	610/644 (94.7)
20% cutoff				
XGB	81/190 (42.6)	20/676 (2.9)	109/190 (57.4)	656/676 (97.1)
LR	88/151 (58.3)	47/715 (6.6)	63/151 (41.7)	668/715 (93.4)

**Figure 6:**
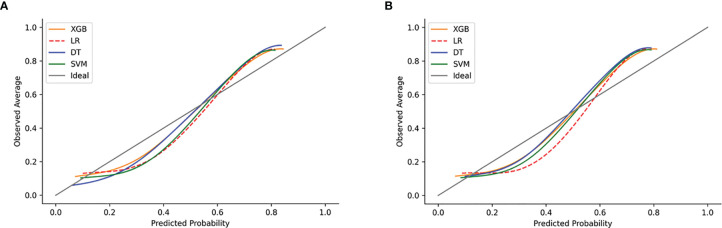
Examples of calibration plots for predicting LNI with various models: XGB, SVM, DT, and LR. **(A)** The training set, and **(B)** the test set. The 45° straight line on each graph represents the perfect match between the observed (*y*-axis) and predicted (*x*-axis) survival probabilities. A closer distance between two curves indicates greater accuracy.

The calibration plots of the two case sets indicated that the predictive probabilities against observed LNI rates showed excellent concordance in the XGB model, followed by the SVM and DT models, respectively. The calibration of the MLR model tended to underestimate the LNI risk across the entire range of predicted probabilities compared with the other two models (**Figure 6**).

**Figure 7:**
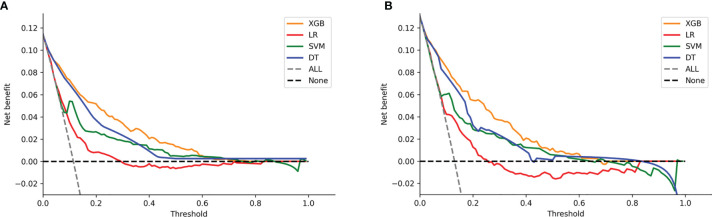
Decision curve analysis graph showing the net benefit against threshold probabilities based on decisions from model outputs. Three curves were obtained based on predictions of the four different models, and the two curves obtained were based on two kinds of extreme decisions. The curves referred to as “All” represent the prediction that all the patients would progress to LNI, and the curves referred to as “None” represent the prediction that all the patients were nLNI. **(A)** The training set, and **(B)** the test set.

DCA of the four models was subsequently constructed in our study (**Figure 7**). The *y*-axis of the decision curve represents the net benefit which is a decision-analytic measure to judge whether any particular clinical decision results in more benefit than harm. Each point on the *x*-axis represents a threshold probability that differentiates between those patients with and without LNI (LNI *vs.* nLNI). This shows that all the models achieved net clinical benefit against a treat-all-or-none plan. With a risk threshold of less than 80%, the ML models showed a greater net benefit for patient interventions in the test set than the MLR model, and the XGB model had the highest net benefit across the whole range of threshold probabilities.

### Model Validation

**Figure 8:**
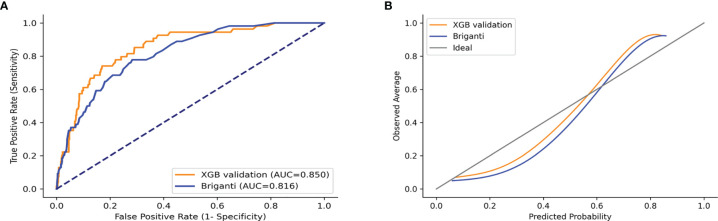
ROC curves and calibration plots of XGB and Briganti nomogram for the validation set. **(A)** ROC curves. **(B)** Calibration plots.

The validation set was used for model validation and the clinical and pathological characteristics of the validation sets are detailed in **Table 4**. In addition, ROC curves and calibration plots were constructed to compare the accuracy of our XGB model and the Briganti nomogram. According to ROC curves, the accuracy of the XGB model is higher than that of the nomogram (AUC: 0.850 *vs.* 0.816) (**Figure 8A**). Moreover, the calibration plots indicated that the XGB model has better consistency across the 0% to 60% range of prediction probability. Instead, the calibration of the nomogram tended to underestimate the LNI risk in the same range (**Figure 8B**).

**Table 4:** Clinical and pathological characteristics of the test and validation sets.

Variables	Validation set		Test set		*p*-value
	NLNI (n = 481) (%)	LNI (n = 54) (%)	NLNI (n = 753) (%)	LNI (n = 113) (%)	
Age at diagnosis					0.006
<62	198 (41.2)	23 (42.6)	362 (48.1)	61 (46.0)	
≥62	283 (58.8)	31 (57.4)	391 (51.9)	52 (54.0)	
Race					0.316
Black	52 (10.8)	7 (13.0)	99 (13.2)	15 (13.4)	
Other	27 (5.6)	4 (7.4)	37 (4.9)	8 (7.0)	
White	402 (83.6)	43 (79.6)	617 (81.9)	90 (79.6)	
Marital status					0.683
Unmarried	53 (11.1)	2 (3.7)	79 (10.5)	16 (14.2)	
Married	428 (88.9)	52 (96.3)	674 (89.5)	97 (85.8)	
T stage					0.154
T1	0 (0.0)	0 (0.0)	0 (0.0)	0 (0.0)	
T2	299 (62.2)	7 (13.0)	458 (60.8)	12 (10.6)	
T3	182 (37.8)	47 (87.0)	291 (38.7)	96 (84.9)	
T4	0 (0.0)	0 (0.0)	4 (0.5)	5 (4.5)	
Tumor size					<0.001
0~10 mm	75 (15.6)	0 (0.0)	104 (13.8)	3 (2.7)	
10~20 mm	249 (51.8)	16 (29.6)	344 (45.7)	22 (19.5)	
20~30 mm	101 (21.0)	22 (40.8)	186 (24.7)	39 (34.5)	
>30 mm	56 (11.6)	16 (29.6)	119 (15.8)	49 (43.3)	
PSA before biopsy					0.063
0~4 ng/ml	69 (14.3)	3 (5.5)	82 (10.9)	6 (5.3)	
4~10 ng/ml	312 (64.9)	25 (46.3)	495 (65.7)	43 (38.1)	
10~20 ng/ml	70 (14.6)	13 (24.1)	139 (18.5)	29 (25.7)	
>20 ng/ml	30 (6.2)	13 (24.1)	37 (4.9)	35 (30.9)	
Primary biopsy Gleason score					0.402
≤6	59 (12.3)	1 (1.9)	112 (14.9)	5 (4.4)	
7	328 (68.2)	24 (44.4)	446 (59.2)	50 (44.2)	
8	50 (10.4)	12 (22.2)	127 (16.9)	29 (25.7)	
≥9	44 (9.1)	17 (31.5)	68 (9.0)	29 (25.7)	
Positive biopsy percentage					<0.001
0~25%	151 (31.4)	12 (22.2)	233 (30.1)	9 (8.0)	
25~50%	218 (45.3)	22 (40.8)	279 (37.1)	37 (32.7)	
50~75%	73 (15.2)	4 (7.4)	145 (19.3)	26 (23.0)	
75~100%	39 (8.1)	16 (29.6)	96 (13.5)	41 (36.3)	

## Discussion

In this study, we developed a more accurate model to predict the risk of LNI in patients with PCa by combining eight clinicopathologic parameters. To our knowledge, this is the first ML model for predicting LNI established by using big data and readily available clinicopathological parameters.

LNI is found in up to 15% of PCa patients upon postoperative pathological examination and is associated with the recurrence and prognosis of PCa (28). As a standard treatment for PCa patients with LNI, ePLND can accurately help diagnose occult micrometastases, allowing PCa patients to get effective treatment and also identifying a more accurate stage of the disease. This is important for postsurgical follow-up and the subsequent selection of adjuvant and salvage therapies (29, 30). However, the rate of detection of an earlier stage or the presence of low-risk tumors at the time of diagnosis rises with PSA screening which leads to a higher rate of surgeries in patients with low- and intermediate-risk tumors. In addition, the likelihood of finding postsurgery-positive LNs decreases. Our study found that the detection rate of postsurgical positive LN was lower than that reported in the literature, at approximately 12% (28). This indicated that some patients were overtreated, resulting in complications such as pelvic lymphocele, ileus, thrombosis, scrotal swelling, nerve injury, and so on (31, 32).

Therefore, determining the LN stage of PCa in patients before surgery is critical in determining whether they should receive ePLND. As routine imaging examinations such as CT scans and MRIs are currently ineffective at detecting nodal metastases (33), and there are only a few promising biomarkers in the preclinical stages of PCa (12, 13), ePLND is the only accurate way of detecting nodal metastases. In order to weigh the benefits and drawbacks of ePLND, the National Comprehensive Cancer Network (NCCN) and the European Association of Urology (EAU) guidelines both recommend the use of nomograms, such as Briganti, MSKCC, and Partin nomograms. These nomograms are largely based on clinicopathologic characteristics that can be easily acquired in the actual clinical procedure (34, 35). These together with several clinicopathologic characteristics which can be readily acquired during the clinical procedure may predict the risk of LNI before surgery. Because of the convenience and practicality of nomograms, they are the most commonly used tools for LNI prediction. However, the performance of these old version nomograms is not always reliable, with a prediction accuracy below 80% (36), and the new version nomograms are less used due to the acquisition of some non-conventional variables (37). Hence, a more advanced prediction model based on the basic variables is needed. XGB is a gradient boosting algorithm that is one of the most powerful techniques for constructing prediction models, and it has been widely used in various medical studies (38, 39).

We found that the five independent risk factors identified by the MLR model were identical to the top 5 most important factors calculated by the XGB model, including T stage, tumor size, PSA before biopsy, GS, and PPC. GS is an evaluation method for determining the state of differentiated PCa tumor cells, and a higher score represents a less differentiated tumor. This parameter is highly correlated with the aggressiveness of the malignancy, and highly aggressive tumors progress more rapidly and are usually associated with LN metastasis. In our study, the XGB model showed that the weight value of GS was the highest, showing the importance of this parameter and indicated that it contributed most to the results obtained (40, 41). As our results demonstrated, the XGB model assigned weighted values to all variables and arranged them by order of importance, thus allowing for more variables to be involved in the analysis and helping physicians to better understand the risk factors.

In this study, the predictive accuracy of our XGB model was the highest in both training and test sets (AUC = 0.908 and 0.883, respectively). Compared to this, the MLR model was less accurate in both training and test sets (AUC = 0.769 and 0.763, respectively). The XGB model was also more accurate than the Briganti nomogram in the validation set (AUC: 0.850 *vs.* 0.816). Previous studies have shown that the predictive accuracy of the Briganti nomogram was 0.798, which was close to our result (0.798 and 0.816) (42). Considering that the nomogram is a visualization tool used in the MLR model, it is established from the MLR algorithm, and this result was not surprising. In addition, the calibration plots for the validation set indicated that the XGB model has a better consistency across the 0% to 60% range of prediction probability. In actual clinical practice, physicians are more concerned with accurately identifying LNI in low-risk PCa patients. It is exciting that our model has better consistency within the low-risk range, and conversely, the nomogram tends to underestimate the risk of LNI in this range. This indicates that our model is better to avoid omissions within the low-risk range. Our results indicate that the MLR model has a weakness in its accuracy when analyzing the linkages seen in multiple data, whereas the XGB model excels at accurately predicting outcomes from multiple unrelated datasets.

Other researchers have tried to use ML to predict LNI. Hou et al. used an ML algorithm combined with mpMRI to build a model for predicting lymph node metastasis in PCa patients. It had a very high prediction accuracy (AUC = 0.906), but the sample size was small and its practical use might be limited as mpMRI was not an easily available parameter (43). Instead, our model was established using a more advanced algorithm based on a big sample and using basic parameters. Similar results were obtained from the calibration plots in both the test and validating sets, which predicted probabilities against observed average LNI rates, indicating that the XGB model had excellent consistency with the MLR model. In addition, the XGB AUC curve obtained here was closer to the ideal line. The MLR model tended to underestimate the LNI risk across the entire range of predicted probabilities, indicating that the use of nomograms based on the MLR model might result in a higher false-negative rate and lead to some LNI patients being omitted.

The significance of determining a cutoff value for a predictive model is to guide the physician during clinical decisions, but it also implies that a certain number of patients below that cutoff value may be missed. The current EAU guidelines suggest that the indication for ePLND is based on the risk of LN metastasis >5% using the Briganti nomogram (44). Using this cutoff, our model could spare about 50% (428/866) of ePLND and only 1.2% (5/428) of patients would be missed. Compared with the MLR model, XGB had a higher positive predictive value (34.5% *vs.* 27.1%) and a negative predictive value (98.9% *vs.* 97.5%), but the percentage of overtreated patients was lower (65.5% *vs.* 72.9%). In addition, our model had low false-negative rates in all the 5%–20% cutoff values. Choosing 20% as the cutoff value can largely reduce the number of ePLND and the possibility of missing patients would be as low as 2.9%. These results imply that our model has a low missing rate, and more LNI patients would be identified. This is consistent with the results of previous studies (44).

Considering that physicians focus on different goals in different situations during clinical practice, DCA was developed by Vickers and Elkin to evaluate clinical effectiveness. The method uses the net benefits at different thresholds to evaluate the clinical utility (45). In our study, within a threshold of 5% to 20%, the net benefit of our XGB model was higher than the other models, which indicates that the value of benefits (i.e., the correct identification of LNI) minus the drawbacks (i.e., overtreatment and omissions) is the biggest.

However, this study still has several limitations. Firstly, the model is based on the SEER database which collects data from the North American population, so there may be gaps in population applicability, necessitating the use of broader populations in future studies. Secondly, tumor size as determined by preoperative imaging was not available for our data and this could cause some errors. We will further improve our model with more complete external validation data in future studies. Thirdly, we excluded patients with <10 LNs examined so as to avoid patients treated with PLND. This is not the ideal definition of ePLND.

This is the first model to predict LNI in PCa patients based on standard clinicopathological features and a novel AI algorithm. Our model performed exceptionally well in predicting LNI in presurgical PCa patients and could potentially assist clinicians to make more accurate and personalized medical decisions. The practical use of this model would be to help surgeons predict the probability of LNI in PCa patients and so help guide surgical alternatives. For patients, it could make some who are more at high risk to be more vigilant, thereby improving their prognosis of PCa.

## Conclusions

We established an ML model based on big data for predicting LNI in PCa patients. Our model had excellent predictive accuracy and practical clinical utility, which might help guide the decision of the urologist and help patients to improve their long-term prognosis. This study follows the trend toward precision medicine for all future patients.

## Data Availability Statement

The datasets presented in this study can be found in online repositories. The software packages of AI algorithms are available at Scikit-learn (https://scikit-learn.org/stable/) and matplotlib (https://matplotlib.org/).The names of the repository/repositories and accession number(s) can be found in the article/**Supplementary Material**.

## Ethics Statement

The studies involving human participants were reviewed and approved by The Medical Ethics Committee at Jinan University’s First Affiliated Hospital. Written informed consent for participation was not required for this study in accordance with the national legislation and the institutional requirements.

## Author Contributions

YZ, LW, and YH conceived and designed the study. YZ and LC provided administrative support. LW, ZC, and XQ collected and assembled the data. YH, LW, HL, and LC analyzed and interpreted the data. All authors contributed to the article and approved the submitted version.

## Funding

This study was supported by the Natural Science Foundation of China (81902615) and the Leading Specialist Construction Project-Department of Urology, the First Affiliated Hospital, Jinan University (711006).

## Conflict of Interest

The authors declare that the research was conducted in the absence of any commercial or financial relationships that could be construed as a potential conflict of interest.

## Publisher’s Note

All claims expressed in this article are solely those of the authors and do not necessarily represent those of their affiliated organizations, or those of the publisher, the editors and the reviewers. Any product that may be evaluated in this article, or claim that may be made by its manufacturer, is not guaranteed or endorsed by the publisher.
